# Multiscale examination of cytoarchitectonic similarity and human brain connectivity

**DOI:** 10.1162/netn_a_00057

**Published:** 2018-11-01

**Authors:** Yongbin Wei, Lianne H. Scholtens, Elise Turk, Martijn P. van den Heuvel

**Affiliations:** Department of Complex Trait Genetics, Center for Neurogenomics and Cognitive Research, Vrije Universiteit Amsterdam, Amsterdam, The Netherlands; Brain Center Rudolf Magnus, Department of Psychiatry, University Medical Center Utrecht, Utrecht University, Utrecht, The Netherlands; Department of Complex Trait Genetics, Center for Neurogenomics and Cognitive Research, Vrije Universiteit Amsterdam, Amsterdam, The Netherlands; Brain Center Rudolf Magnus, Department of Psychiatry, University Medical Center Utrecht, Utrecht University, Utrecht, The Netherlands; Brain Center Rudolf Magnus, Department of Psychiatry, University Medical Center Utrecht, Utrecht University, Utrecht, The Netherlands; Brain Center Rudolf Magnus, Department of Neonatology, Wilhelmina Children’s Hospital, University Medical Center Utrecht, Utrecht University, Utrecht, The Netherlands; Department of Complex Trait Genetics, Center for Neurogenomics and Cognitive Research, Vrije Universiteit Amsterdam, Amsterdam, The Netherlands; Brain Center Rudolf Magnus, Department of Psychiatry, University Medical Center Utrecht, Utrecht University, Utrecht, The Netherlands; Department of Clinical Genetics, Amsterdam UMC, Vrije Universiteit Amsterdam, Amsterdam Neuroscience, Amsterdam, The Netherlands

**Keywords:** Connectivity, Network, Graph theory, BigBrain, Cytoarchitectonic differentiation, Structural type

## Abstract

The human brain comprises an efficient communication network, with its macroscale connectome organization argued to be directly associated with the underlying microscale organization of the cortex. Here, we further examine this link in the human brain cortex by using the ultrahigh-resolution BigBrain dataset; 11,660 BigBrain profiles of laminar cell structure were extracted from the BigBrain data and mapped to the MRI based Desikan–Killiany atlas used for macroscale connectome reconstruction. Macroscale brain connectivity was reconstructed based on the diffusion-weighted imaging dataset from the Human Connectome Project and cross-correlated to the similarity of laminar profiles. We showed that the BigBrain profile similarity between interconnected cortical regions was significantly higher than those between nonconnected regions. The pattern of BigBrain profile similarity across the entire cortex was also found to be strongly correlated with the pattern of cortico-cortical connectivity at the macroscale. Our findings suggest that cortical regions with higher similarity in the laminar cytoarchitectonic patterns have a higher chance of being connected, extending the evidence for the linkage between macroscale connectome organization and microscale cytoarchitecture.

## Author Summary

The human brain connectome organization has been suggested to associate with cytoarchitecture similarity. Here, we utilize the state-of-the-art ultrahigh-resolution BigBrain dataset and diffusion-weighted imaging dataset to examine this association. Our results show that cortical regions with higher cytoarchitecture similarity are more likely to be connected, as well as connected by stronger white matter tracts. This work further extends our understanding of the interaction between macroscale cortico-cortical connectivity organization and microscale cortical cytoarchitecture.

## INTRODUCTION

The human brain connectome is a comprehensive map comprising interconnections of neural elements at multiple scales (Sporns, 2011; Sporns, Tononi, & Kötter, 2005). At the microscale, neuron-to-neuron connections are formed by axons, dendrites, and synapses, processing and transmitting neural information by means of electrical and chemical signals (Cossell et al., 2015; Ullo et al., 2014; Yuste, 2011). In parallel, the macroscale connectome consists of cortical regions that are linked by large-scale white matter tracts, providing a structural backbone supporting functional specialization and efficient information integration (Bullmore & Sporns, 2012; van den Heuvel & Sporns, 2013).

In recent years, studies have aimed to bridge these two levels of brain organization, and have suggested that macroscale brain connectivity might be indeed associated with cortical cytoarchitecture patterns. The large variety in cytoarchitecture across the human cerebral cortex (Brodmann, 1909; von Economo & Koskinas, 1925) has been suggested to yield a rich body of cortical circuit patterns for diverse functions, with, for example, larger and more spinous pyramidal cells observed in prefrontal cortex compared with primary regions (e.g., visual cortex; Elston, 2003; Elston, Benavides-Piccione, & DeFelipe, 2001). Expanding the thoughts of cytoarchitectonic variation, studies have further proposed that the interareal cytoarchitectonic differentiation plays a pivotal role in shaping cortico-cortical connections (Barbas, 2015). Moreover, modern human and animal connectome studies have also provided quantitative evidence for the relation between brain connectivity and cortical cytoarchitecture. Highly connected cortical regions have layer III pyramidal cells with large basal dendritic tree size, a large number of spines per neuron in the macaque brain (Scholtens, Schmidt, de Reus, & van den Heuvel, 2014), and large neuron soma size in the human brain (van den Heuvel, Scholtens, Barrett, Hilgetag, & de Reus, 2015). The presence or absence of interregional connectivity has also been observed to be associated with the cortical cytoarchitectonic differentiation, showing that regions with more similar cytoarchitecture type had a greater chance to be connected (Beul, Barbas, & Hilgetag, 2017; Beul, Grant, & Hilgetag, 2015; Goulas et al., 2016; Goulas, Uylings, & Hilgetag, 2017; Hilgetag, Medalla, Beul, & Barbas, 2016).

Recently, an ultrahigh-resolution three-dimensional model of a cell body–stained human brain was provided by Amunts et al. (2013); 7,404 histological sections were collected from a complete paraffin-embedded brain at a nearly cellular resolution of 20 micrometers, resulting in the BigBrain dataset. The BigBrain provides higher sampling rate within the whole brain and allows direct mapping to the modern neuroimaging data. Therefore, it can serve as a good quantitative reference to link macroscale in vivo brain mapping findings to microscale cytoarchitecture in the human cerebral cortex. In the current study, we used the BigBrain dataset and sought to examine how the cortical cytoarchitecture shapes the large-scale connectivity in human brain. We combined the information on cortical layer composition extracted from the BigBrain images with the diffusion-weighted imaging (DWI) data from the Human Connectome Project (Van Essen et al., 2013). Linking BigBrain profile similarity to the reconstructed macroscale connectome, we extend evidence for the association between microscale cytoarchitectonic similarity and macroscale connectome organization in the human brain.

## METHODS

### BigBrain Data

As described in detail by Amunts et al. (2013), the BigBrain data includes 7,404 histological sections with 20-μm thickness that were cut in coronal plane from a complete postmortem paraffin-embedded human brain of a 65-year-old man without any neurological or psychiatric diseases in clinical records. All sections were stained for cell bodies (Merker, 1983) and were digitized into high-resolution images of 13,000 × 11,000 pixels (10 × 10 μm^2^). The digitized images were downsampled to 20 × 20 μm^2^ to obtain an isotropic resolution matching the slice thickness of 20 mm. Defects of histological artifacts, such as rips, tears, folds, missing and displaced pieces, distortion (shear), stain inhomogeneity, and crystallization, were repaired both manually and automatically to restore the integrity of all slices (Amunts et al., 2013). All preprocessed images were downloaded in PNG format from http://bigbrain.loris.ca/main.php.

#### BigBrain profiles.

**Figure 1.**
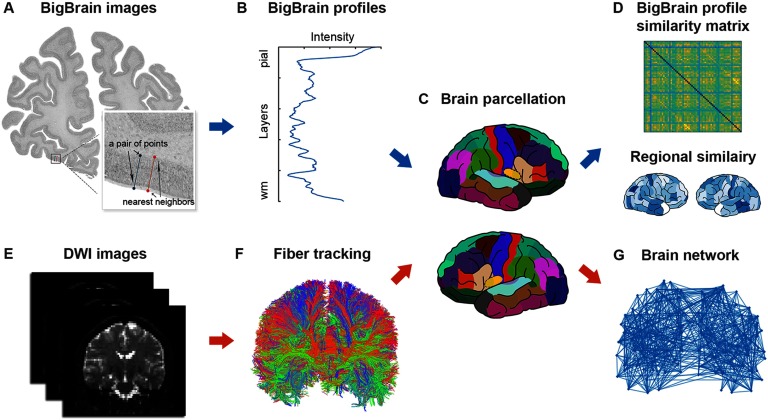
Overview of the data processing steps. (A) An example of a BigBrain image, selected pair of points, and its closest neighbor pair. (B) A manual BigBrain profile was extracted from the BigBrain image according to randomly selected points. (C) BigBrain profile was registered to the Desikan–Killiany atlas and averaged within each cortical region. (D) The average BigBrain profiles were correlated between every two cortical regions to obtain a similarity matrix. In parallel, we used DWI data (E) and performed fiber tracking (F) to reconstruct the brain structural network (G). BigBrain profile similarity was linked to properties of the brain structural network.

Using the BigBrain dataset, we extracted cortical profiles that delineate the laminar cell number and density of the cortex. Resulting BigBrain profiles were extracted according to the following steps. First, 11,660 pairs of points on the cortex boundaries were manually selected from BigBrain images. During the selection, random image sections were chosen and four pairs of points were selected with short intervals. For each pair of points, the first point was selected on the pial surface and the second on the white matter surface (Figure 1A), forming a line segment perpendicular to the two surfaces. Second, we searched the closest neighbor pair for each pair of points according to the Euclidean distance. Linking each pair of points and their nearest neighbor formed a quadrilateral in the image that covered a small cortical area. Inside the quadrilateral, we uniformly sampled 1,000 blocks from the pial surface to the white matter surface. The mean image intensity of each block was computed to reflect the level of cell size and density within the block (because images were cell body stained). A curve of image intensities was subsequently generated for each pair of points and named the BigBrain profile (Figure 1B). The resulting BigBrain profiles were found to be comparable with profiles based on the histological information derived from the von Economo and Koskinas atlas; see Supporting Information, Wei, Scholtens, Turk, & van den Heuvel, 2018). All procedures were implemented in MATLAB.

Using the BigBrain dataset, we extracted cortical profiles that delineate the laminar cell number and density of the cortex. Resulting BigBrain profiles were extracted according to the following steps. First, 11,660 pairs of points on the cortex boundaries were manually selected from BigBrain images. During the selection, random image sections were chosen and four pairs of points were selected with short intervals. For each pair of points, the first point was selected on the pial surface and the second on the white matter surface (Figure 1A), forming a line segment perpendicular to the two surfaces. Second, we searched the closest neighbor pair for each pair of points according to the Euclidean distance. Linking each pair of points and their nearest neighbor formed a quadrilateral in the image that covered a small cortical area. Inside the quadrilateral, we uniformly sampled 1,000 blocks from the pial surface to the white matter surface. The mean image intensity of each block was computed to reflect the level of cell size and density within the block (because images were cell body stained). A curve of image intensities was subsequently generated for each pair of points and named the BigBrain profile (Figure 1B). The resulting BigBrain profiles were found to be comparable with profiles based on the histological information derived from the von Economo and Koskinas atlas; see Supporting Information, Wei, Scholtens, Turk, & van den Heuvel, 2018). All procedures were implemented in MATLAB.

#### Anatomical registration.

BigBrain profiles were registered to a common space of MRI data for further region-wise analyses. First, BigBrain images (i.e., PNG files) were transformed to NIfTI format, forming a customized BigBrain image, and resampled to a resolution of 400 μm to optimize computation time. Second, we registered the BigBrain image to the reference brain volume in the Montreal Neurological Institute (MNI) International Consortium of Brain Mapping (ICBM) 152 space (downloaded from https://bigbrain.loris.ca/), by applying the affine registration tool FLIRT (Jenkinson & Smith, 2001; Jenkinson, Bannister, Brady, & Smith, 2002) followed by a nonlinear registration using FNIRT tools (Andersson, Jenkinson, & Stephen, 2007), employing a b-spline representation of the registration warp field (Supporting Information Figure S1, Wei et al., 2018). Third, brain parcellations in the FreeSurfer fsaverage template were affine registered to the MNI ICBM 152 space by using FLIRT, followed by warping to the customized BigBrain image by using the inversed registration warp field generated in the second step (Supporting Information Figure S1, Wei et al., 2018). Brain parcellations included (a) the 68-region Desikan-Killiany (DK) atlas (Desikan et al., 2006; Fischl et al., 2004), which was used in the main results, and (b) the 114-region DK subdivision (Cammoun et al., 2012), and (c) the von Economo–Koskinas (EK) atlas (Scholtens, de Reus, & van den Heuvel, 2015; Scholtens, de Reus, de Lange, Schmidt, & van den Heuvel, 2016; von Economo & Koskinas, 1925) for validation purposes. Fourth, given the coordinates of the point pairs (which were used to extract BigBrain profiles) in the BigBrain images, we searched for their nearest voxels in the customized BigBrain image. This way, each BigBrain profile was assigned to the DK region that its nearest voxel belongs to, making it possible to obtain the regional BigBrain measurements in the original BigBrain space; 2,438 BigBrain profiles located too far from the nearest cortical voxel (>1.5 interquartile range [IQR] distance to the nearest cortical voxel) were excluded as outliers. Four hundred thirteen BigBrain profiles crossing DK region boundaries—with the nearest neighbor point-pair located in a different DK region—were further excluded to avoid samples at boundaries. As a result, 8,809 BigBrain profiles were included in further analyses.

### BigBrain Profile Similarity

A regional BigBrain profile was obtained per region by averaging BigBrain profiles within the region. A mean number of 133 profiles (standard deviation [*SD*]: 158) was included per DK region. Eight DK regions with fewer than 20 profiles were excluded from further analyses because of the possible bias resulting from an insufficient number of samples. We noted that adopting additional thresholds of 0 and 50 profiles to examine the possible effect of small regions revealed similar findings (see Supporting Information, Wei et al., 2018). Next, a matrix of interregional similarity of BigBrain profiles was obtained by calculating the Pearson’s correlation between the regional BigBrain profiles of every pair of DK cortical areas. The resulting profile similarities were observed to demonstrate a nonnormal distribution and were transformed to a normal distribution with mean of 1 and *SD* of 0.2 by matching ranks. Using the raw similarity (i.e., nonredistributed data) showed similar results (see Supporting Information, Wei et al., 2018). For regional analysis, values of each column within the matrix were averaged, resulting in a vector representing the mean similarity level of a region to the rest of the brain.

### MRI Data

High-quality T1-weighted MRI data and diffusion-weighted MRI data from 215 subjects (age [mean ± *SD*]: 29.8 ± 3.4 years old) from the Q3 data release of the Human Connectome Project were used in the current study (Van Essen et al., 2013). The FreeSurfer software package (Fischl, 2012) was used to obtain brain tissue segmentation and cortical mantle reconstruction from the T1-weighted data. The DK atlas was used for cortical parcellation (Desikan et al., 2006; Fischl et al., 2004).

White matter tracts were reconstructed from DWI images by using the following procedure (van den Heuvel, Scholtens, de Reus, & Kahn, 2016). First, the 18 sets of b = 0 volumes were averaged, and the 270 diffusion images were realigned and corrected for small head motions and common gradient-induced distortions (Andersson & Skare, 2002). Second, the diffusion profile within each voxel was reconstructed using generalized q-sampling imaging, allowing for reconstruction of crossing fibers (Yeh, Wedeen, & Tseng, 2010). Third, deterministic tractography was performed to reconstruct white matter tracts, performing fiber assignment by using the Fiber Assignment by Continuous Tracking (FACT) algorithm (Mori, Crain, Chacko, & van Zijl, 1999). For each voxel, eight streamline seeds were started and tracking was stopped if the streamline reached a voxel of low preferred diffusion direction (fractional anisotropy < 0.1), exited the gray matter/white matter mask, or made a sharp turn (>45°).

### Connectome Construction

A structural network was constructed from the set of reconstructed tractography streamlines and the cortical parcellation for each subject. Here, network nodes were defined as cortical regions, and edges were placed between nodes that were connected by reconstructed streamlines. The number of reconstructed streamlines (NOS) was used to weight network edges. NOS was transformed to a normal distribution (mean = 1, *SD* = 0.2). Moreover, streamline density of each edge was obtained by dividing NOS by the mean volume of the two connected regions, and also used as a connection weight. A group-averaged binary network was formed by placing an edge between two brain regions if those regions were connected in more than 50% of the subjects. Alternative thresholds of >40% and >60% were used as validations (see Supporting Information, Wei et al., 2018). The weighted network was obtained by averaging nonzero weights (i.e., NOS and streamline density) of each edge in the group binary network across all subjects.

### Connectome Analyses

Graph theoretical analyses were conducted on the reconstructed structural network. Four nodal metrics were calculated on both the group binary and the group-weighted networks, including nodal degree/strength (for the binary/weighted network, respectively), betweenness centrality, clustering coefficient, and mean path length. First, degree was computed for each node as the number of edges connected to a node. Likewise, nodal strength was obtained by taking the sum of weights of edges connected to a node. Second, betweenness centrality was calculated as the fraction of all shortest paths in the network that traverse a given node, where the shortest path length was defined as the lowest number of edges that must be traversed to go from one node to the other. The obtained betweenness centrality values were found to be nonnormally distributed and were thus log transformed. Third, the clustering coefficient for each node was computed as the proportion of edges between the node’s neighbors divided by the number of edges that could possibly exist between these neighbors. Finally, the mean path length was obtained for each node by averaging the shortest path length between this node and the rest of the nodes in the network. Graph metrics were computed using the brain connectivity toolbox (https://sites.google.com/site/bctnet/; Rubinov & Sporns, 2010).

### Statistical Analysis

A two-tailed two-sample *t* test was used to investigate the difference of BigBrain profile similarity between interconnected cortical regions and nonconnected cortical regions. Pearson’s correlation analysis was performed to estimate the association of BigBrain profile similarity with connection weights in the group-weighted structural network. Analyses were reperformed for connections within left and right hemisphere, and between hemispheres, separately. Moreover, regional BigBrain profile similarity was correlated to the nodal degree/strength, clustering coefficient, path length, and betweenness centrality of the group-averaged connectome map. We also conducted a partial correlation between the regional BigBrain profile similarity level and nodal graph metrics by taking the number of individual BigBrain profiles per region as covariates to investigate the influence of the sample size of BigBrain profiles. To examine the dependency among graph metrics, multiple linear regression was performed as:

$$Y=\beta_{0}+\beta_{1}X_{1}+\beta_{2}X_{2}+\beta_{3}X_{3}+\beta_{4}X_{4}+\varepsilon$$

with *Y* indicating the BigBrain profile similarity and *X*_1_, …, *X*_4_ nodal strength, betweenness centrality, clustering coefficient, and shortest path length, respectively, and with *β*_i_ representing coefficients and *ε* the residuals. For all above analyses, effects reaching a false discovery ratio (FDR) corrected *q* < 0.05 (across all 28 tests done in the main result) were taken as significant.

### Interregional Distance

As discussed by recent studies (Beul et al., 2017, 2015), interregional distance may play an important role in the organization of brain connectivity. Here, we also examined whether the physical distance between brain regions influenced BigBrain profile similarity and its association with connectivity. First, we calculated the coordinates of centroids of each DK region in the FreeSurfer fsaverage template by averaging the (X, Y, Z) coordinates of all voxels within a region. Second, the Euclidean distance between centroids of DK regions was computed and taken as the interregional physical distance. Third, connections were divided into three categories according to the distance ranking, including short-range (top 25% shortest connections), long-range (top 25% longest connections), and mid-range connections (others). The BigBrain similarity difference across three connectivity categories was examined using one-way ANOVA analysis and post hoc two-sample *t* tests. Finally, interregional distance, together with the mean cortical volume and surface area (i.e., the mean volume and surface area between every cortical region pair), were regressed out from both BigBrain profile similarity and connectivity strength, separately, by using linear regression. Residuals were used to reevaluate the association of BigBrain profile similarity with connectivity.

### Validation Analyses Using von Economo–Koskinas Data

The EK data was used to examine the agreement of our BigBrain profiles with classical measurements. In 1925, Constantin von Economo and George Koskinas published a comprehensive brain atlas comprising 48 “most important” distinct cortical areas as well as detailed layer-specific histological information on neuronal count, neuron size, and cortical thickness (von Economo & Koskinas, 1925). A digital version of the EK atlas based on the FreeSurfer Software (Fischl, 2012) was used to link the historical histology data to modern anatomical imaging (Scholtens et al., 2015, 2016). We mapped BigBrain profiles to the EK atlas and computed the regional averaged mean and *SD* of BigBrain profiles. Additionally, the length of the line segment formed by each pair of points was recorded as an assessment of cortical thickness. In parallel, EK profiles were generated for each EK area by sampling 1,000 steps from the pial to the white matter surface and for each step assigning the corresponding layers’ (neuron density × neuron size) value (von Economo & Koskinas, 1925). The mean and *SD* of the resulting EK profiles were also computed. Pearson’s correlation was used to estimate the similarity of BigBrain profile with EK profiles and the agreements of properties (i.e., mean and *SD*) of the two types of profiles (see Supporting Information, Wei et al., 2018).

## RESULTS

**Figure 2.**
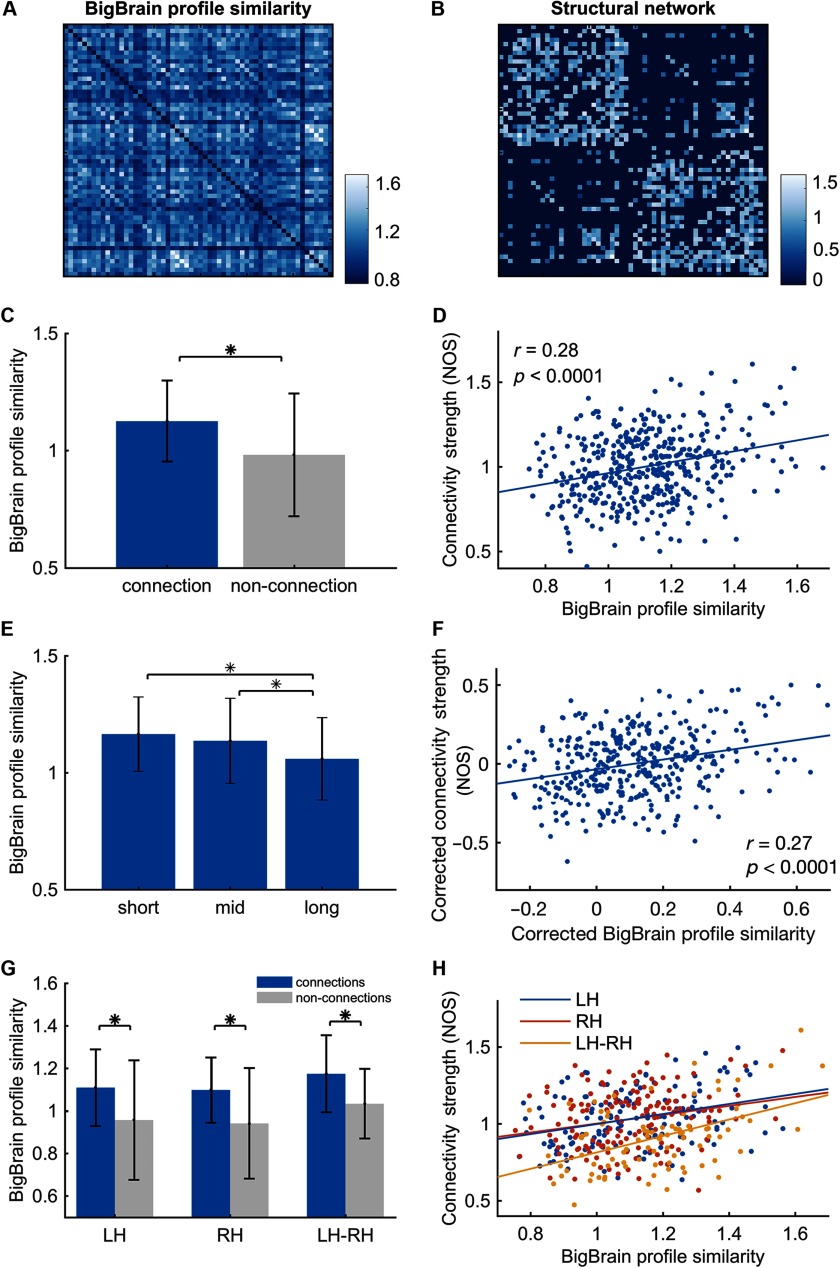
Association of BigBrain profile similarity with structural connectivity at the edge level. (A) BigBrain profile similarity matrix. (B) Group-weighted structural connectivity matrix. (C) BigBrain profile similarity between interconnected regions was significantly higher than between nonconnected regions (*t* = 9.36, *p* < 0.0001). (D) BigBrain profile similarity was positively correlated with connection weight (NOS) of the structural network (*r* = 0.28, *p* < 0.0001). (E) BigBrain profile similarity was different among short-, mid-, and long-range connections (*F*_(*df* = 389)_ = 9.75, *p* < 0.0001). Short-range connections showed significantly higher profile similarity than mid- (*t* = 3.42, *p* = 0.0007) and long-range connections (*t* = 4.34, *p* < 0.0001). (F) Regressing out interregional distance and the mean regional volume and surface area, BigBrain profile similarity still correlated with the connectivity strength (*r* = 0.27, *p* < 0.0001). (G) Both intra- and interhemispheric BigBrain profile similarity was higher between connected regions compared with nonconnected regions (Left hemisphere [LH]: *t* = 8.31, *p* < 0.0001; right hemisphere [RH]: *t* = 9.74, *p* < 0.0001 interhemisphere [LH-RH]: *t* = 7.53, *p* < 0.0001). (H) Taking LH, RH, and LH-RH connections separately, BigBrain profile similarity consistently showed correlations with connection strength (*r* = 0.32, 0.23, and 0.47, separately, all *p* < 0.0001; *significant differences).

BigBrain profile similarity between interconnected cortical regions was observed to be significantly higher than between nonconnected regions (*T*_(*df* = 1,709)_ = 9.36, *p* < 0.0001, FDR corrected; Figure 2C), suggesting that regions with higher cytoarchitectonic similarity were more likely to be connected. With respect to all connected regions, BigBrain profile similarity was significantly correlated with connection strength of the group structural network (NOS: *r* = 0.28, *p* < 0.0001; streamline density: *r* = 0.17, *p* = 0.0003, FDR corrected; Figure 2D), which indicates that cortical regions showing higher cytoarchitectonic similarity have a higher probability of being linked by stronger white matter connections.

Considering the interregional distance, we observed a significant difference for BigBrain profile similarity across short-, mid-, and long-range connections (*F*_(*df* = 389)_ = 9.75, *p* < 0.0001, FDR corrected), showing the highest similarity for short-range connections and the lowest for long-range connections (Figure 2E). Notably, BigBrain profile similarity showed correlations with connectivity strength for each subset of connections: short-range (*r* = 0.30, *p* = 0.0032), mid-range (*r* = 0.16, *p* = 0.0245), and long-range connectivity (*r* = 0.32, *p* = 0.0014, all FDR corrected). We further regressed out this effect, together with the effect of cortical area size, from the BigBrain profile similarity and reevaluated the observed association of profile similarity to cortico-cortical connectivity. Residuals of BigBrain profile similarity remained to be larger between interconnected regions than nonconnected regions (*T*_(*df* = 1,709)_ = 10.14, *p* < 0.0001, FDR corrected). The correlation between profile similarity and connectivity strength also persisted (*r* = 0.27, *p* < 0.0001, FDR corrected; Figure 2F).

The association between BigBrain profile similarity and connectivity was further examined in context of intra- and interhemispheric connections. BigBrain profile similarity was found to be higher in connected regions than nonconnected regions within the left hemisphere (*T*_(*df* = 376)_ = 5.37, *p* < 0.0001, FDR corrected) and right hemisphere (*T*_(*df* = 433)_ = 7.19, *p* < 0.0001, FDR corrected), as well as between interhemisphere connected and nonconnected regions (*T*_(*df* = 838)_ = 7.54, *p* < 0.0001, FDR corrected; Figure 2G). Taking within-hemisphere and interhemisphere connections separately, BigBrain profile similarity consistently showed a significant correlation with the connection strength (*r* = 0.32 and 0.23 for connections in left and right hemisphere, and *r* = 0.47 for interhemispheric connections, all *p* < 0.0001, FDR corrected; Figure 2H).

**Figure 3.**
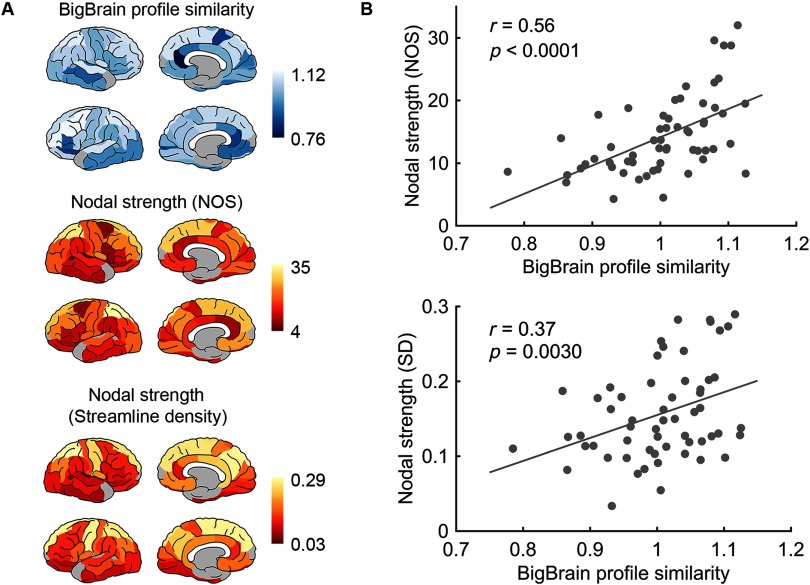
(A) The pattern of regional BigBrain profile similarity (top) and nodal strength (middle: NOS weights; bottom: streamline density weights). (B) Regional BigBrain profile similarity showed significant correlation with nodal strength (top: NOS, *r* = 0.56, *p* < 0.0001; bottom: streamline density, *r* = 0.37, *p* = 0.0030).

Correlating the pattern of regional BigBrain profile similarity to nodal strength of the group structural network showed a significant correlation (NOS: *r* = 0.56, *p* < 0.0001; streamline density: *r* = 0.37, *p* = 0.0042, FDR corrected; Figure 3), confirming that regions with higher BigBrain profile similarity to the rest of regions tend to be connected by stronger connections at the macroscale level. Correlation analysis between regional BigBrain profile similarity and nodal degree of the group binary network demonstrated similar results (*r* = 0.52, *p* < 0.0001, FDR corrected). Findings persisted when we examined the partial correlation between BigBrain profile similarity and macroscale nodal degree/strength by taking the number of BigBrain profiles of each cortical region as covariates (*ρ* = 0.45, *p* = 0.0004 for the strength; *ρ* = 0.38, *p* = 0.0035 for the degree, FDR corrected). Regressing out the interregional distance from BigBrain profile similarity and connectivity strength (NOS) revealed a similar correlation (*r* = 0.41, *p* = 0.0014, FDR corrected).

**Figure 4.**
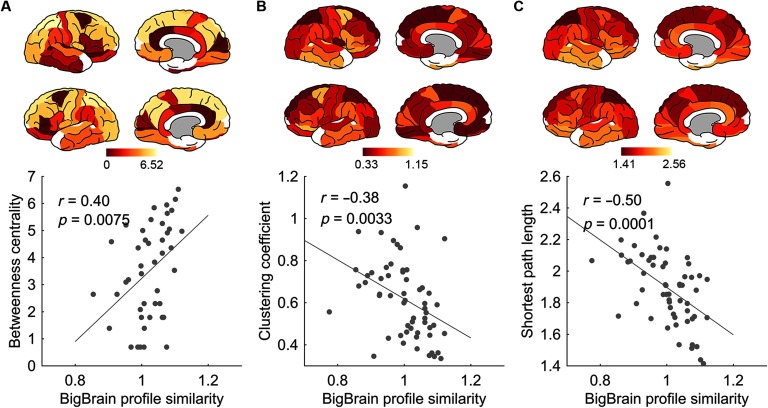
The association of regional BigBrain profile similarity with (A) betweenness centrality (*r* = 0.40, *p* = 0.0075), (B) clustering coefficient (*r* = −0.38, *p* = 0.0033), and (C) mean shortest path length (*r* = −0.50, *p* = 0.0001) of the group-weighted network (NOS weights).

Regional BigBrain profile similarity was significantly correlated with betweenness centrality (NOS,*r* = 0.44, *p* = 0.0033, FDR corrected; Figure 4). Meanwhile, negative associations were found with clustering coefficient (*r* = −0.35, *p* = 0.0070, FDR corrected) and mean shortest path length (*r* = −0.50, *p* = 0.0001, FDR corrected; Figure 4), indicating that regions with more similar cytoarchitectonic patterns with the rest of brain were less locally clustered and more globally connected to the rest of the network. Analyzing all metrics together in a multiple linear regression showed a significant effect for nodal strength (*p* = 0.0182, FDR corrected), but not for other graph metrics (*p* = 0.2578, 0.1103, and 0.7363 for betweenness centrality, clustering coefficient, and mean shortest path length, respectively), indicating that the effect of the other graph metrics is largely dependent on the effect of nodal strength.

## DISCUSSION

In this study, we investigated how cortical cytoarchitecture differentiation was associated with macroscale connectome organization. Data from the BigBrain project was used to extract laminar cytoarchitecture profiles across the entire cerebral cortex. BigBrain profile similarity for cortico-cortical connections was found to be significantly higher than for nonconnections, indicating that regions with higher similarity in laminar cytoarchitectonic patterns are more likely to be connected. Furthermore, the pattern of regional BigBrain profile similarity was strongly correlated with the pattern of nodal strength, clustering coefficient, and shortest path length of the structural network, suggesting that cortical regions with higher cytoarchitectonic similarity tend to be linked by stronger white matter connections and to be more involved in global integration. Taken together, our findings suggest that microscale cortical cytoarchitecture similarity is closely associated with macroscale brain connectome organization.

The use of cytoarchitecture profiles to quantify variation in human cerebral cortical architecture has a long history (Haug, 1956; Mackey & Petrides, 2009; Schleicher, Morosan, Amunts, & Zilles, 2009; Schleicher, Zilles, & Wree, 1986; Schmitt & Böhme, 2002; Wree, Schleicher, & Zilles, 1982). In addition to classical histological methods, cytoarchitectonic profiles as provided by the BigBrain dataset allow for investigation of gradual changes in the volume fraction of cell bodies from pial surface to white matter surface, rather than concentrating on a particular cell type of a single layer (Amunts & Zilles, 2015). A rich body of literature examining the difference in profile shape between adjacent blocks within the cortical ribbon has provided strong evidence for the position of interareal borders (Caspers et al., 2013; Kujovic et al., 2013; Schleicher et al., 2000; Schleicher, Amunts, Geyer, Morosan, & Zilles, 1999; Schleicher et al., 2009). Moreover, findings have also shown that vertically oriented cortical columns have similar laminar patterns of cell types and cell densities (Zilles & Amunts, 2010). Here, we used regional averaged BigBrain profiles to represent the intra-area laminar cytoarchitectonic pattern. Our validations revealed that the regional BigBrain profile features were associated with the areal characteristics of neuronal size and density proposed by von Economo and Koskinas (see Supporting Information, Wei et al., 2018), further indicating BigBrain profiles as a good representation of the microscale cytoarchitectonic features.

It has been eloquently reported that the presence of long-range cortico-cortical connection is associated with the cortical cytoarchitectonic patterning of the human and animal cortex. Cortical regions with more similar cytoarchitecture types have been suggested to have a larger chance to be connected, leading to the predictive structural model of cortico-cortical connections (Barbas, 2015). The structural model has been broadly verified in human prefrontal regions (Barbas, Hilgetag, Saha, Dermon, & Suski, 2005), visual system of the cat (Hilgetag & Grant, 2010), as well as the macaque (Beul et al., 2017), cat (Beul et al., 2015), mouse (Goulas et al., 2017), and human cortex (Goulas et al., 2016). Further zooming in on neuronal morphology, macroscale highly connected cortical regions have been found to show a large pyramidal complexity in layer III, quantified among other things by a large basal dendritic tree size and a large number of spines per neuron in the macaque brain (Scholtens et al., 2014), and a large neuronal soma size in the human brain (van den Heuvel et al., 2015). Disruption in connectivity in brain disorders, such as schizophrenia, has also been observed to be associated to alterations in layer III pyramidal spine density (van den Heuvel et al., 2016) and in vivo cortical cytoarchitectonic disruptions (Wei et al., 2018). Here, our findings demonstrate that strongly connected cortical regions show higher BigBrain profile similarity in the human brain cortex than regions with no connections. Compared with a prior study of the human brain that examined cytoarchitectonic similarity and connectivity (Goulas et al., 2016), the current study extends investigations by using the comprehensive BigBrain data with high spatial sampling rates and continuous descriptions of profiles from the pial to the white matter surface. Consistent findings across studies together support the structural model hypothesis of associated microscale cortical cytoarchitectonic organization and macroscale cortico-cortical connectivity in the mammalian brain (Barbas, 2015; Barbas et al., 2005; Beul et al., 2017, 2015; Goulas et al., 2016, 2017; Hilgetag & Grant, 2010).

The neurobiological mechanism underlying the observed association between cytoarchitectonic similarity and cortico-cortical connectivity remains to be determined. A probable explanation has been provided in the context of brain ontogenesis, which argues that the development of cortical cytoarchitecture is associated with the establishment of connections (Barbas, 2015; Beul et al., 2017, 2015; Goulas et al., 2016, 2017). According to this hypothesis, the variation of laminar structure within the cerebral cortex arises during development, differentiating limbic areas with a relatively short developmental path combined with a poorly defined cortical layers from the longer developing association areas with well-delineated layer structures (Barbas & García-Cabezas, 2016). Cortical regions with similar laminar cytoarchi tecture patterns may thus develop during a similar time window (Barbas, 2015; Beul et al., 2017, 2015; Goulas et al., 2016, 2017). Furthermore, the formation of cortico-cortical connections has been argued to be shaped by the cortical cytoarchitecture development. Evidence has shown that connections originating from neurons in the early developed limbic cortices terminated in layer I of other areas (Barbas, 2015), known to be one of the earliest formed layers of the neocortex (Marin-Padilla, 1970). Together, the revealed association between cortical cyto architecture similarity and cortico-cortical connectivity may reflect the overlap in time windows of their development.

A number of remarks have to be made when interpreting the findings of our study. First, it is noted that regional difference in cortical volume could be of influence on the here reported association. We therefore performed additional analyses using streamline density as connection weight to correct for regional cortical volume, as well as a partial correlation analysis taking the number of BigBrain profiles per region as covariates (because larger regions will have more profiles). Both analyses showed results similar to the main analysis. Additionally, changing the region exclusion threshold to 0 or 50 BigBrain profiles revealed similar correlations, further suggesting that small regions with relative few profile samples had no specific effect on the results. Second, the BigBrain data was obtained from a single 65-year-old male donor. As a result, our analysis did not take into account possible individual variability, gender differences, and aging effects on profile similarity (Shaw et al., 2008; Zilles et al., 1997). Studies constructing a microscale reference brain based on a larger dataset would be of particular interest. Third, reconstruction of the macroscale connectome was limited by the current techniques of in vivo MRI. Diffusion imaging relies on water diffusion as an indirect probe of axon geometry, which has well-known limitations with respect to the reconstruction of complicated pathways (Jbabdi & Johansen-Berg, 2011). High-field imaging, acquisition of more diffusion directions, and application of advanced white matter pathway reconstruction protocols may result in better detection of complicated fibers.

This study uses the state-of-the-art BigBrain dataset to obtain comprehensive cortical cytoarchitecture profiles of the human brain and shows an association of laminar profile similarity with anatomical network organization. Findings provide new evidence for a potential interplay between microscale cortical cytoarchitecture organization and macroscale cortico-cortical connectivity, which provide insights into the neurobiological mechanisms underlying the macroscale brain connectome. Understanding the cross-modal interaction between the micro- and macroscale of human brain organization may pave a new avenue for unraveling neuropathology in neurological and psychiatric disorders involving disruptions on both ends of the scale.

## ACKNOWLEDGMENTS

We thank Marcel A. de Reus for his help with data processing. We appreciate Maurits Ridder for his contribution in collecting data. Diffusion weighted imaging data were kindly provided in part by the Human Connectome Project, WU-Minn Consortium (Principal Investigators: David van Essen and Kamil Ugurbil; 1U54MH091657) funded by the NIH Institutes and Centers that support the NIH Blueprint for Neuroscience Research; and by the McDonnell Center for Systems Neuroscience at Washington University.

## AUTHOR CONTRIBUTIONS

Yongbin Wei: Investigation; Methodology; Writing – original draft; Writing – review & editing. Lianne H. Scholtens: Writing – original draft; Writing – review & editing. Elise Turk: Data curation; Writing – original draft; Writing – review & editing. Martijn P. van den Heuvel: Conceptualization; Funding acquisition; Investigation; Project administration; Supervision; Writing – review & editing.

## FUNDING INFORMATION

Martijn P. van den Heuvel, Nederlandse Organisatie voor Wetenschappelijk Onderzoek (http://dx.doi.org/10.13039/501100003246), Award ID: VIDI-452-16-015; Nederlandse Organisatie voor Wetenschappelijk Onderzoek, Award ID: ALWOP.179, and a Fellowship of MQ. Yongbin Wei, China Scholarship Council, Award ID: 201506040039.

## TECHNICAL TERMS

Connectome: The wiring diagram between neural elements of a nervous system.
White matter tracts: Bundles connecting gray matter areas to each other, mainly consisting of myelinated axons.
Cytoarchitecture: The cellular structure of the nervous system’s tissues under the microscope.
BigBrain: A freely accessible ultrahigh-resolution 3D digital atlas of the human brain.
Diffusion-weighted imaging: An MRI technique measuring the diffusion of water molecules to reconstruct white matter connections of the brain.
Brain parcellation: The spatial division of brain into a set of nonoverlapping regions.
FreeSurfer: A brain imaging software package for MRI data analysis.
