# Supplementary material for: Multiscale examination of cytoarchitectonic similarity and human brain connectivity
Source: Netw Neurosci. 2018 Nov 1;3(1):124–37. doi: 10.1162/netn_a_00057 (PMC6372019; doi:10.1162/netn_a_00057)
Supplement: Supplementary file 1 [file netn-03-124-s001.pdf]

Wei, Y., Scholtens, L. H., Turk, E., & van den Heuvel, M. P. (2018). Supporting Information for "Multiscale examination of cytoarchitectonic similarity and human brain connectivity." *Network Neuroscience*, *3*(1), 124–137. https://doi.org/10.1162/netn_a_00057

Supplemental Materials

Validations using alternative processing strategies

Results were validated by adopting different processing strategies, including applying the 114-region subdivision DK atlas, setting the region inclusion threshold to 0 and 50 profiles, and generating the group average network at a threshold of 40% and 60% prevalence.

First, using the 114-region subdivision DK atlas, the BigBrain profile similarity between inter-connected cortical regions was found to be consistently higher than between non-connected regions ($t = 12.5$, $p = 3 \times 10^{-35}$). The pattern of regional BigBrain profile similarity was also reliably correlated with both the pattern of nodal strength of the group weighted network (NOS: $r = 0.38$, $p = 0.0001$) and nodal degree of the group binary network ($r = 0.35$, $p = 0.0004$). These findings suggested that our main results were not driven by the selection of cortical parcellations.

Second, setting the region inclusion threshold to 0 (meaning that all regions were included) and 50 profiles (meaning that only regions with more than 50 profiles were included), BigBrain profile similarity for connections was significantly higher than for non-connections ($t = 12.2$, $p = 3 \times 10^{-33}$ for regions with >0 profile, $t = 8.9$, $p = 3 \times 10^{-18}$ for regions with >50 profiles). Significant correlations were also found between the pattern of regional BigBrain profile similarity and nodal strength ($r = 0.55$, $p < 0.0001$ for regions with >0 profile, $r = 0.54$, $p = 0.0001$ for regions with >50 profiles) and degree ($r = 0.52$, $p < 0.0001$ for regions with >0 profile, $r = 0.48$, $p = 0.0008$ for regions with >50 profiles), indicating the exclusion of small regions didn't change the nature of our results.

Third, thresholding the group structural network at 40% or 60% revealed similar findings: a significantly higher BigBrain profile similarity was found for connections as compared to non-connections (40%: $t = 10.6$, $p = 1 \times 10^{-25}$, 60%: $t = 10.2$, $p = 6 \times 10^{-24}$), and a significant correlation was found between the pattern of BigBrain profile similarity and nodal strength

(40%: r = 0.56, p < 0.0001, 60%: r = 0.55, p < 0.0001) and degree (40%: r = 0.52, p < 0.0001, 60%: r = 0.51, p < 0.0001). These results indicated that different structural network density didn't alter our main results.

Validations using the raw correlation coefficients

The raw correlation coefficient between BigBrain profiles was taken as the measurement of similarity to examine the influence of the performed normal-distribution transformation. We consistently found significant differences of BigBrain profile similarity between interconnected cortical regions and non-connected regions ($T_{(df = 1709)} = 7.64$, $p < 0.0001$), as well as the significant correlation of profile similarity with connection strength (NOS: $r = 0.22$, $p < 0.0001$). Moreover, the pattern of regional BigBrain profile similarity was also associated with the nodal degree ($r = 0.41$, $p = 0.0013$) and strength ($r = 0.45$, $p = 0.0003$, NOS weighted), confirming that our main results were not affected by the normal-distribution transform.

Validating BigBrain profiles using von Economo-Koskinas data

We assessed the agreement of BigBrain profiles with the cytoarchitectonic data derived from von Economo and Koskinas (EK) atlas (von Economo and Koskinas, 1925). BigBrain profiles were registered to the FreeSurfer-based EK atlas (Scholtens et al., 2016) and averaged within each EK region. In parallel, information of laminar layer thickness, neuron cell size, and neuron density of each area were taken from the EK atlas to generate EK profiles describing the level of [cell size × density] from pial surface to white matter surface. EK profiles were additionally resampled to 1000 discrete steps to match the BigBrain profiles (Fig. S2). We correlated BigBrain profiles with the EK profiles for each EK region and observed significant associations between the two types of profiles ($r = 0.52 \pm 0.17$, ranging

from 0.24 to 0.83 for all EK areas), indicating the BigBrain profiles to be comparable with the laminar cytoarchitecture in classic EK atlas.

Furthermore, cortical thickness estimates derived from BigBrain profiles were also found to be significantly correlated to Von Economo-Koskinas's measures of thickness across cortical regions ($r = 0.61$, $p = 0.0005$, FDR corrected) (Fig. S2), indicating that the co-registration process of BigBrain profiles in the current study yielded output which provided consistent measurement of cortical morphology. The regional averaged mean and SD of BigBrain profiles showed significant correlations with the regional mean and SD of EK profiles across all cortical regions ($r = 0.51$, $p = 0.0082$ and $r = 0.57$, $p = 0.0024$, for the mean and SD, respectively, FDR corrected) (Fig. S3), suggestive of the consistency in cortical cytoarchitectonic patterns.

Examining effects of DK area size

In order to examine the effects of DK area size on our results, we first obtained the mean cortical volume and surface area for each DK region, by averaging across all subjects in the HCP dataset. The cortical volume and surface area were observed to be correlated with the regional BigBrain profile similarity (volume: $r = 0.50$; surface area: $r = 0.53$; both ps < 0.0001) and connectivity degree (volume: $r = 0.69$; surface area: $r = 0.74$; both ps < 0.0001)/strength (volume: $r = 0.75$; surface area: $r = 0.80$; both ps < 0.0001). Next, we regressed out the cortical volume and surface area, separately, from both the regional BigBrain profile similarity and nodal degree/strength using the linear regression. Residuals after the regression were used to re-perform the correlation. We observed a decreased effect of correlations between regional profile similarity and nodal degree/strength, but the significance still held mostly (volume: $r = 0.29$, $p = 0.0165$, for degree; $r = 0.31$, $p = 0.0106$, for strength; surface area: $r = 0.23$, $p = 0.0592$, for degree; $r = 0.25$, $p = 0.0423$, for strength).

These findings suggested that the association of BigBrain profile similarity pattern with nodal degree/strength was not entirely driven by the DK area size.

Within-region profile heterogeneity

In the current study, distinct numbers of BigBrain profiles were extracted from cortical regions and averaged within the same region, resulting in the consideration of potential effects of within-region profile heterogeneity and "smoothness" caused by averaging profiles. We thus performed three additional analyses to examine whether these effects played a role in our main findings.

First, we calculated the Kendall's coefficient of concordance (KCC) for profiles of each cortical region to represent the within-region profile homogeneity. KCC was computed as follows:

$$W = \frac{\sum(R_\mathrm{i})^2 - N\bar{R}^2}{\frac{1}{12}K^2(N^3 - N)}$$

where W is the KCC within each cortical region, ranged from 0 to 1; R_i the sum rank of the ith depth level; $\bar{R}$ the mean of R_i; K the number of profiles within a cortical region; and N the number of ranks (i.e., depth levels; here, $N = 1000$). The resultant regional KCC ranged from 0.22 to 0.67 with mean ± SD of 0.51 ± 0.09 (Fig. S4A). The pattern of KCC didn't correlate with the pattern of BigBrain profile similarity across the cortex ($r = -0.07$, $p = 0.55$), nor with the pattern of regional connectivity strength ($r = 0.06$, $p = 0.63$) (Fig. S4B), suggesting that variations of within-region profile heterogeneity didn't drive the observed association between BigBrain profile similarity and cortico-cortical connectivity.

Second, we performed a permutation test to examine whether within-region profile homogeneity is higher than the homogeneity of randomly selected profiles. We randomly shuffled the DK region label for all profiles and computed KCC within the randomly

assigned regions. The average KCC of all regions was recorded. The computation was permutated 10,000 times to generate a null distribution of KCC. Comparing the original averaged KCC (i.e., 0.51) to the null distribution revealed a significance level of $p < 0.0001$ (Fig. S4C), indicating that within-region profile heterogeneity is significantly higher than within randomly selected profiles.

Third, we randomly chose 20 BigBrain profiles from profiles of each cortical region and averaged these profiles to obtain a region-wise BigBrain profile. Selecting 50 profiles showed similar results (data not shown). In this manner, the same number of BigBrain profiles was included to generate the regional profile, ruling out the potential effect of different sizes of profile samples. We repeated the randomization 10,000 times and consistently found a higher BigBrain similarity between connected regions than non-connected regions (T ranged from 7.18 ~ 12.16, mean ± SD = 9.88 ± 0.55, all ps < 0.0001) (Fig. S4D). Furthermore, the pattern of regional BigBrain profile similarity reliably showed correlations with the pattern of connectivity degree (r ranged from 0.17 – 0.57, mean ± SD = 0.38 ± 0.05, more than 99.1% randomizations showed $p < 0.05$) and strength (r ranged from 0.21 – 0.61, mean ± SD = 0.42 ± 0.05, more than 99.9% randomizations showed $p < 0.05$) (Fig. S4E), suggesting that these findings were not affected by the distinct “smoothness” levels derived from different profile sample sizes.

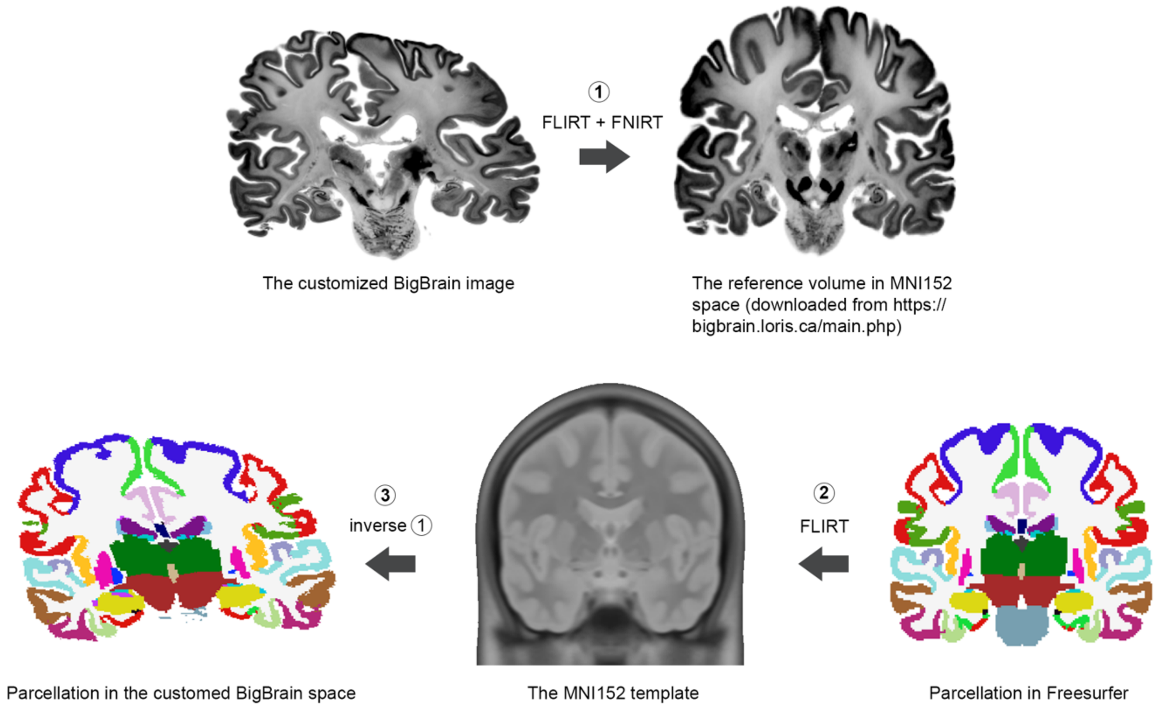

Figure S1. Schematic representation of the co-registration process. (1) BigBrain image was registered to the reference brain volume in the MNI ICBM 152 space (downloaded from https://bigbrain.loris.ca), by applying the FSL FLIRT and FNIRT tools (https://fsl.fmrib.ox.ac.uk). (2) Brain parcellations in the FreeSurfer fsaverage template were affine registered to the MNI ICBM 152 space using FLIRT, followed by (3) warping to the BigBrain image space using the inversed registration warp field generated in (1).

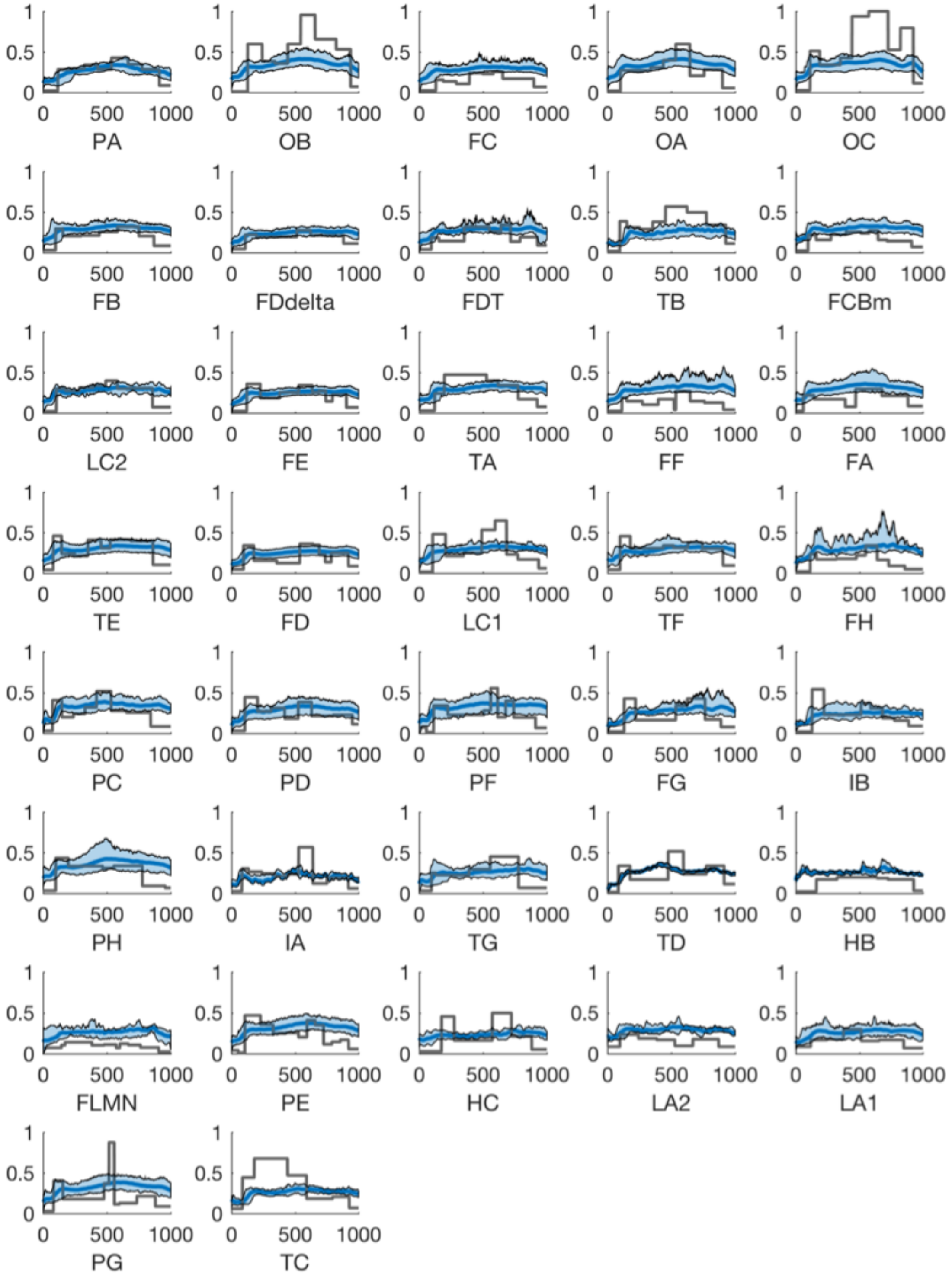

Figure S2. BigBrain profiles and EK profiles of all EK areas. Areas were ranked according to the correlation between BigBrain profiles and EK profiles in a descending order. Dark blue: regional BigBrain profiles; Dark grey: EK profiles; Shaded region: lower and upper 95% confidence intervals.

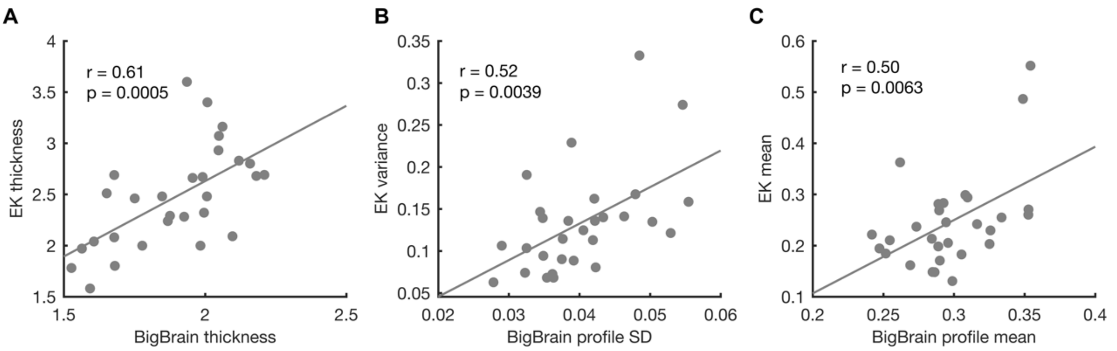

Figure S3. Linking BigBrain profiles to Von Economo-Koskinas (EK) data. (A) Cortical thickness estimates in BigBrain data were correlated with thickness measurements in EK atlas (r = 0.61, p = 0.0005). (B) The mean and (C) standard deviation (SD) of BigBrain profiles were correlated with the mean (r = 0.50, p = 0.0063) and variance (r = 0.52, p = 0.0039) of [neuron size × neuron density] in EK atlas.

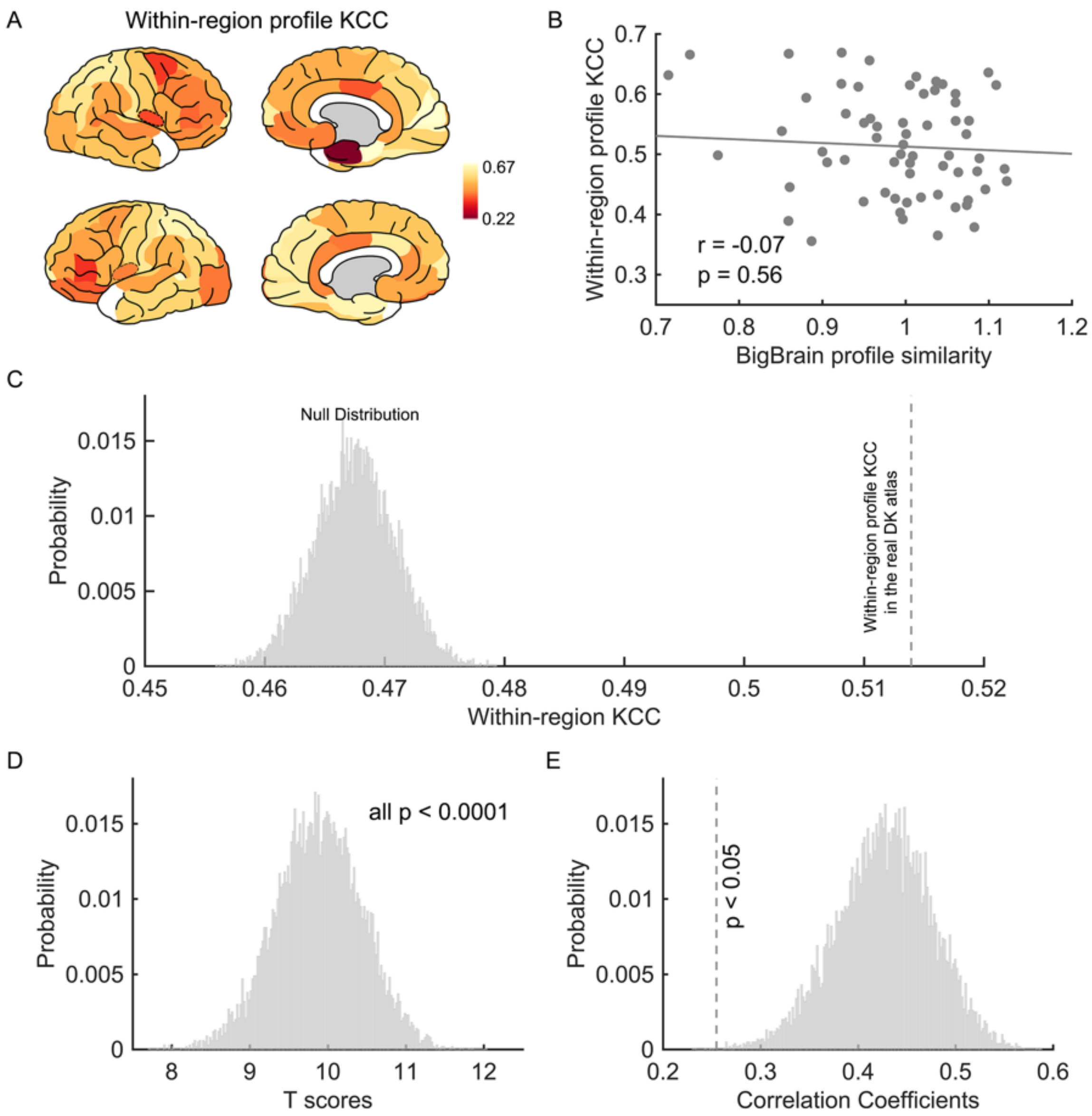

Figure S4. (A) The pattern of within-region profile KCC. (B) The pattern of within-region KCC did not correlate with the pattern of BigBrain profile similarity (r = -0.07, p = 0.56). (C) The mean within-region KCC was significantly higher than the null conditions generated by randomly reassigned DK label to BigBrain profiles (p < 0.0001, 10,000 permutations). (D) Histogram of t scores generated by 10,000 randomizations, where 20 profiles were randomly collected within each region to generate the mean regional profiles and t tests were performed for profile similarity between connections and non-connections. p < 0.0001 was observed for all randomizations. (E) Histogram of r scores generated by 10,000 randomizations, where 20

profiles were randomly collected within each region to generate the mean regional profiles and correlation analyses were performed between the pattern of profile similarity nodal strength.

Reference

Scholtens, L.H., de Reus, M.A., de Lange, S.C., Schmidt, R., van den Heuvel, M.P., 2016. An MRI Von Economo – Koskinas atlas. NeuroImage. https://doi.org/10.1016/j.neuroimage.2016.12.069

von Economo, C.F., Koskinas, G.N., 1925. Die cytoarchitektonik der hirnrinde des erwachsenen menschen. Julius Springer Verlag, Berlin.
